# A ubiquitous bone marrow reservoir of preexisting SARS-CoV-2-reactive memory CD4^+^ T lymphocytes in unexposed individuals

**DOI:** 10.3389/fimmu.2022.1004656

**Published:** 2022-10-04

**Authors:** Jinchan Li, Simon Reinke, Yu Shen, Lena Schollmeyer, Yuk-Chien Liu, Zixu Wang, Sebastian Hardt, Christian Hipfl, Ute Hoffmann, Stefan Frischbutter, Hyun-Dong Chang, Tobias Alexander, Carsten Perka, Helena Radbruch, Zhihai Qin, Andreas Radbruch, Jun Dong

**Affiliations:** ^1^ Cell Biology, Deutsches Rheuma-Forschungszentrum Berlin (DRFZ), Institute of the Leibniz Association, Berlin, Germany; ^2^ Berlin Brandenburg Center for Regenerative Therapies (BCRT), Charité - Universitätsmedizin Berlin, Corporate Member of Freie Universität Berlin, Humboldt-Universität zu Berlin, and Berlin Institute of Health, Berlin, Germany; ^3^ Center for Musculoskeletal Surgery, Charité - Universitätsmedizin Berlin, Corporate Member of Freie Universität Berlin, Humboldt-Universität zu Berlin, and Berlin Institute of Health, Berlin, Germany; ^4^ Schwiete-Laboratory for Microbiota and Inflammation, Deutsches Rheuma-Forschungszentrum Berlin (DRFZ), Institute of the Leibniz Association, Berlin, Germany; ^5^ Institute of Allergology, Charité - Universitätsmedizin Berlin, corporate member of Freie Universität Berlin, Humboldt-Universität zu Berlin, and Berlin Institute of Health, Berlin, Germany; ^6^ Allergology and Immunology, Fraunhofer Institute for Translational Medicine and Pharmacology (ITMP), Berlin, Germany; ^7^ Department of Rheumatology and Clinical Immunology, Charité - Universitätsmedizin Berlin, Corporate Member of Freie Universität Berlin, Humboldt-Universität zu Berlin, and Berlin Institute of Health, Berlin, Germany; ^8^ Institute of Neuropathology, Charité - Universitätsmedizin Berlin, Corporate Member of Freie Universität Berlin, Humboldt-Universität zu Berlin, and Berlin Institute of Health, Berlin, Germany; ^9^ Medical Research Center, The First Affiliated Hospital of Zhengzhou University, Zhengzhou, China

**Keywords:** SARS-CoV-2, cross-reactive, memory CD4^+^ T lymphocytes, human bone marrow, peripheral blood, polyfunctional, tissue-resident memory T cells (Trm)

## Abstract

Circulating, blood-borne SARS-CoV-2-reactive memory T cells in persons so far unexposed to SARS-CoV-2 or the vaccines have been described in 20-100% of the adult population. They are credited with determining the efficacy of the immune response in COVID-19. Here, we demonstrate the presence of preexisting memory CD4^+^ T cells reacting to peptides of the spike, membrane, or nucleocapsid proteins of SARS-CoV-2 in the bone marrow of all 17 persons investigated that had previously not been exposed to SARS-CoV-2 or one of the vaccines targeting it, with only 15 of these persons also having such cells detectable circulating in the blood. The preexisting SARS-CoV-2-reactive memory CD4^+^ T cells of the bone marrow are abundant and polyfunctional, with the phenotype of central memory T cells. They are tissue-resident, at least in those persons who do not have such cells in the blood, and about 30% of them express CD69. Bone marrow resident SARS-CoV-2-reactive memory CD4^+^ memory T cells are also abundant in vaccinated persons analyzed 10-168 days after 1°-4° vaccination. Apart from securing the bone marrow, preexisting cross-reactive memory CD4^+^ T cells may play an important role in shaping the systemic immune response to SARS-CoV-2 and the vaccines, and contribute essentially to the rapid establishment of long-lasting immunity provided by memory plasma cells, already upon primary infection.

## Introduction

Human severe acute respiratory syndrome coronavirus 2 (SARS-CoV-2) causes the coronavirus disease 2019 (COVID-19), which poses a substantial threat to public health. The individual human response against the SARS-CoV-2 varies dramatically, from asymptomatic to severe disease characterized by pneumonia and acute respiratory distress syndrome (ARDS) often resulting in death. Presently, it is unclear why only a few of the infected develop severe COVID-19, and most are protected from it.

One reason may be preexisting cross-reactive immunological memory to SARS-CoV-2. Recent studies have revealed preexisting, cross-reactive T cell immunity to SARS-CoV-2 in unexposed healthy donors and asymptomatic COVID-19 patients. We and others have shown that SARS-CoV-2-reactive T cells could be detected in the peripheral blood (1–7) of more than 20% of healthy individuals who had previously not been exposed to SARS-CoV-2 or a vaccine mimicking it (2–11). A correlation of T cell reactivity towards SARS-CoV-2 spike (S) protein and human common cold corona viruses (HCoVs) (3, 9, 12, 13), suggestive of cross-reactivity, was only observed in unexposed healthy donors but not in COVID-19 convalescents (6, 12). These circulating, preexisting cross-reactive CD4^+^ T cells from unexposed healthy donors had pro-inflammatory Th1 and Tfh-like anti-viral potential (1, 13). Such cells likely contribute to fast and efficient immune responses against SARS-CoV-2 (2–4, 6, 12, 14, 15), and their presence correlates with protection of naïve individuals against SARS-CoV-2 infection in COVID-19 contacts (4).

Circulating memory T cells reactive to a particular antigen are easy to identify, but little is known about their persistence (16) and their contribution to long-term memory. Their exclusive contribution to systemic and tissue-specific immunity is challenged by the increasing realization of the relevance of tissue-resident memory T cells (Trm) (17–19). CD69^+^ Trm have been described (19–22), as well as CD69^-^ Trm (22). In particular, CD4^+^ Trm of the lung mediating local immunity to respiratory viruses (23, 24) and presumably also to SARS-CoV2 (25) have been reported.

We have previously shown that bone marrow provides dedicated niches for the long-term maintenance of immune memory cells (26). Trm of human bone marrow maintain long-term memory to systemic antigens, in particular viruses like measles, mumps, and rubella, even when their circulating counterparts are no longer detectable (19). Following a systemic challenge, bone marrow Trm are rapidly mobilized and then contribute essentially to the secondary immune response (27). Here, we compare preexisting, SARS-CoV-2 cross-reactive memory CD4^+^ T lymphocytes of bone marrow and blood of 17 individuals. Bone marrow of all individuals did contain preexisting, persistent, polyfunctional SARS-CoV-2-reactive memory CD4^+^ T lymphocytes, in numbers largely exceeding those circulating in their blood. At frequencies of above 10^-4^ among memory CD4^+^ T cells, these Trm qualify a significant pillar of preexisting immunity to SARS-CoV-2.

## Materials and methods

### Study subjects

This study was conducted in accordance with the approval from the local ethics committee (Ethikkommission der Charité-Univerisitätsmedizin Berlin; EA1/005/21) and informed consent obtained according to the Declaration of Helsinki. Thirty-one paired bone marrow biopsy (BM) and peripheral blood (PB) samples were collected from anonymous systemically healthy individuals (13 male and 18 female; age ± SEM = 66.52 ± 2.65) undergoing hip replacement surgery at the Charité – Universitätsmedizin Berlin from September 2020 to May 2022. The study cohort consisted of 17 SARS-CoV-2 and the vaccines unexposed donors and 14 vaccinated donors. Samples from donors 1-17 and donors 18-31 were collected before and after the COVID-19 vaccination campaign in Germany (Dec. 2020), respectively. Donors 18-31 had been vaccinated either with or without booster vaccination (since Oct 2021). Donors 1-22 were also validated for levels of SRARS-CoV-2 S- (spike) or N- (nucleocapsid) protein specific antibody titres in plasma isolated from PB samples.

### Sample collection and preparation

BM and PB samples were subject to preparation in less than 4 hours as described previously (19, 28). Briefly, peripheral blood mononuclear cells (PBMCs) were isolated from 5-15 mL blood samples (diluted with room temperature PBS buffer at a 1:1 ratio) by density gradient centrifugation using Ficoll-Paque Plus (Cytiva). Bone marrow mononuclear cells (BMMCs) were filtered through a 70 µM cell strainer prior to density gradient centrifugation using Ficoll-Paque Plus. The resulting plasma layers from PB samples were collected as plasma samples and stored at -80°C until further use. Cell number was determined using a MACSQuant (Miltenyi Biotech).

### Determination of spike- and nucleocapsid-protein specific antibodies by enzyme-linked immunosorbent assay

To evaluate the levels of IgG antibodies specific to spike S1 and nucleocapsid proteins of SARS-CoV-2 in plasma samples, respective human IgG ELISA Kits from Biolegend were used according to the manufacturer’s instructions. In short, plasma samples were tested at serial dilutions of 1:500, 1:1000, 1:2000, 1:4000 and 1:8000, each with two technical replicates. Absorbance was read immediately by Spectramax Plus 384 Microplate Reader at 450 nm. Data were analyzed using the Graphpad Prism 9.1.0.

### Flow cytometry analysis

Staining, data acquisition, and analysis were carried out as described previously (19, 28). Briefly, eight- to twelve- colour flow cytometry analysis was performed for the analysis of cell count, phenotype, and cytokine profile using a MACSQuant Analyzer 10 flow cytometer (Miltenyi Biotec) and LSRFortessa flow cytometer (BD Biosciences). The following anti-human antibodies were used to stain cells in four different panels: 1). propidium iodide (PI), or DAPI; 2). CD3 BV785, CD19 BV510, CD14 PO, CD4 PE-Cy5, CD8 PE, CD45RO BV650, CD154 BV421, IFN-γ PE-Cy7, IL-2 FITC, TNF-α Allophycocyanin, and fixable Live/Dead PO; 3). CD3 BV785, CD4 PE-Cy5, CD14 PO, CD19 BV510, Live/Dead PO, CD45RA BV605, CCR7 A488, CD154 PE, IL-2 APC-Cy7, TNF-α Allophycocyanin, and IFN-γ PE-Cy7; and 4). CD3 BV785, CD4 PE-Cy5, Live/Dead PO, CD14 PO, CD19 BV510, CD45RO BV650, CD154 PE, IL-2 APC-Cy7, TNF-α Allophycocyanin, IFN-γ PE-Cy7, and CFSE. Stained cells were acquired using FACSDiva (BD Biosciences) software and data were analyzed with FlowJo (BD Biosciences).

### Identification of *ex vivo* antigen-reactive memory CD4^+^ T lymphocytes

BMMCs and PBMCs were cultured separately in RPMI 1640 Supplementaled with 1% GlutaMAX, 100 U/mL penicillin, 100 µg/mL streptomycin, and 5% (vol/vol) human AB serum. According to cell count, cells were seeded in an appropriate culture plate at a cell density of 1-2 × 10^7^ cells/mL. Anti-CD28 (1 µg/mL), CMV (38 µg/mL), tetanus toxoid (TT; 1 lethal factor [Lf]/mL), or a mixture of three peptide pools – the spike-, membrane-, and nucleocapsid-protein of SARS-Cov-2 (0.6 nmol of each peptide/mL) were used to stimulate cells. Stimulation with CD28 alone and in combination with Staphylococcus enterotoxin B (SEB; 1µg/ml) were used as negative and positive controls, respectively. For each condition, at least 5-10 × 10^6^ cells were stimulated for a total of 12 h at 37° and 5% CO_2_, with brefeldin A (1 µg/mL) added to the mixture for the last 10 h. Stimulated cells were first stained for surface antigens followed by fixation with 2% paraformaldehyde and permeabilizing solution 2 according to the manufacture’s instruction. Antigen-reactive memory CD4^+^ T cells were identified as Live/Dead^-^CD19^-^CD14^-^CD3^+^CD8^-^CD4^+^CD45RO^+^ (/CD45RA^-^CCR7^+/-^) CD154^+^cytokine^+^. About 2 × 10^6^ lymphocytes were analyzed for each condition.

Alternatively, CD69^+^ BMMCs were isolated using the CD69 MicroBead Kit according to the manufacturer recommendations. Magnetically separated (flow-through) CD69^-^ BMMCs were labelled with carboxyfluorescein succinimidyl ester (CFSE) at the final concentration of 1 µM. The CFSE labelled CD69^-^ BMMCs were then mixed with the CD69^+^ BMMCs and co-cultured for antigenic stimulation under conditions as described above. Antigen-reactive memory CD4^+^ T cells from CD69^-^ and CD69^+^ subsets of BMMCs were identified as Live/Dead^-^CD19^-^CD14^-^CD3^+^CD8^-^CD4^+^CD45RO^+^CFSE^+^/^-^ CD154^+^cytokine^+^.

### Statistics

Statistical analyses were performed with Graphpad Prism software (version 9.1.0). For analysis of paired blood and bone marrow samples, Wilcoxon matched-pairs signed rank test was used. For analysis of unpaired samples, Mann–Whitney U test was used. A *p*-value < 0.05 was considered statistically significant; *p<0.05, **p<0.01, ***p<0.001, and ****p<0.0001.

## Results

### Bone marrow hosts SARS-CoV-2 reactive memory CD4^+^ T cells in both unexposed and vaccinated donors

**Figure 1:**
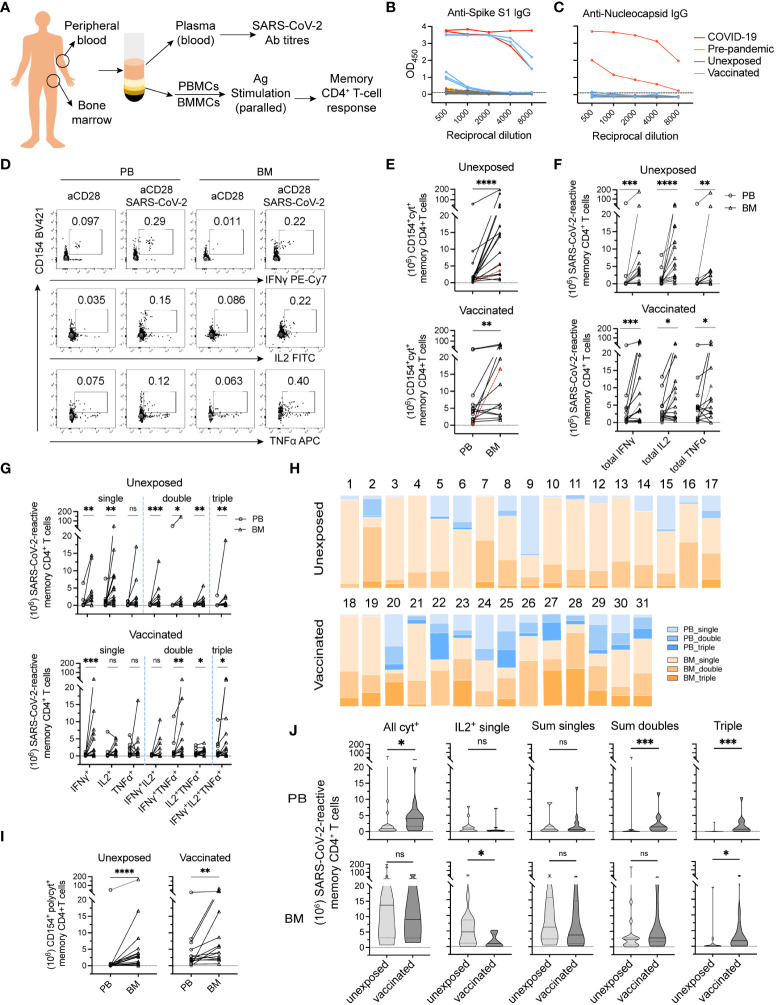
Bone marrow hosts polyfunctional memory CD4^+^ T cells against SARS-CoV-2 in both unexposed and vaccinated adult donors. **(A)** Flowchart of experimental setup. Mononuclear cells isolated from paired peripheral blood (PB) and bone marrow biopsy (BM) samples were collected from adult donors. Plasma from PB samples were collected for measuring IgG titers specific for SARS-CoV-2 Spike and Nucleocapsid proteins. PBMCs and BMMCs were stimulated with S/M/N peptides mix of SARS-CoV-2 and analyzed for antigen-reactive memory CD4^+^ T cell responses. (**B** + **C**) Levels of antibodies to SARS-CoV-2 S **(B)** and N **(C)** proteins were measured using serial dilutions (two technical replicates of each dilution) of donor’s plasma in an Enzyme-Linked Immunosorbent Assay (ELISA). Red line, positive controls (n = 2) from acute phase ICU COVID-19 patients analyzed in previous study (1); yellow lines, negative control samples collected prior to COVID-19 pandemic (Pre-pandemic; n = 2); black lines, samples from unexposed donors (Unexposed; n = 17); blue lines, samples from COVID-19 vaccinated donors (Vaccinated; n = 5). The Y-axis corresponds to the mean optical density (OD) values assessed at 450 nm as depicted reciprocal dilutions on the X-axis. **(D)** Representative plots showing SARS-CoV-2-reactive memory CD4^+^ T cells expressing CD154 and one or more of the induced cytokine production (IL-2, TNF-α, or IFN-γ). **(E–G)** Estimated absolute numbers (29, 30) of CD154^+^cytokine^+^ (**E**; all cytokine producing cells), total individual CD154^+^cytokine^+^ **(F)** and single, double and triple cytokine-producing cells **(G)**. Symbols in red and dashed lines indicate estimated cell numbers of PB calculated according to frequencies under detection limit (10^-4^ of memory CD4^+^ T cells). **(H)** Proportions of single, double, and triple cytokine-producing cells from **(G)** among total antigen-reactive cells **(E)** to the sum of PB and BM from donors analyzed on the individual levels. **(I)** Estimated absolute numbers of polyfunctional cells between paired blood and bone marrow samples. **(J)** Shown are estimated absolute numbers of indicated cell types in unexposed *vs* vaccinated donors. Values are presented as median (thick line) with 25^th^- and 75^th^-percentile. *p<0.05, **p<0.01, ***p<0.001, ****p<0.0001; ns, not significant.

Paired bone marrow and blood samples (**Figure 1A**) were obtained from 31 individuals (aged 35 to 87 years) that had not been infected with SARS-CoV-2. Of those, 17 unexposed (donors 1-17) were examined prior to and 14 vaccinated (donors 18-31) after 1° (donors 18-20), or 10-168 days after 3° (donors 21-28 and 30, 31) or 4° (donor 29) vaccination, respectively (**Table 1**). While the former were seronegative for S (**Figure 1B**) and N (**Figure 1C**) proteins of SARS-CoV-2, the latter were positive for S and negative for N.

**Table 1:** Study subjects.

Donor	Sample	Relative to COVID-19vaccination campaign	Vaccine	Vaccinationstatus	Sex	Age
**1**	Sep.2020	No	─	─	F	43
**2**	Sep.2020	No	─	─	M	35
**3**	Sep.2020	No	─	─	F	55
**4**	Sep.2020	No	─	─	M	77
**5**	Sep.2020	No	─	─	M	67
**6**	Sep.2020	No	─	─	M	66
**7**	Oct.2020	No	─	─	F	61
**8**	Oct.2020	No	─	─	F	53
**9**	Oct.2020	No	─	─	F	48
**10**	Nov.2020	No	─	─	F	57
**11**	Nov.2020	No	─	─	F	75
**12**	Nov.2020	No	─	─	M	85
**13**	Dec.2020	No	─	─	M	40
**14**	Dec.2020	No	─	─	F	80
**15**	Dec.2020	No	─	─	F	78
**16**	June 2021	No	─	─	F	80
**17**	Oct.2021	No	─	─	M	69
**18**	July 2021	Yes	unk	w/o booster	M	63
**19**	July 2021	Yes	unk	w/o booster	M	78
**20**	Oct.2021	Yes	unk	w/o booster	M	58
**21**	Dec.2021	Yes	Moderna	3×, 10 d	M	61
**22**	Dec.2021	Yes	BioNTech	3×, 6 wks	F	79
**23**	Jan.2022	Yes	BioNTech	3×, 9 wks	F	80
**24**	Feb.2022	Yes	Moderna	3×, 9 wks	M	85
**25**	Feb. 2022	Yes	Moderna	3×, 6 wks	F	54
**26**	Feb. 2022	Yes	Moderna	3×, 11 wks	M	74
**27**	Mar. 2022	Yes	Moderna	3×, 15 wks	F	49
**28**	Mar. 2022	Yes	BioNTech	3×, 17 wks	F	87
**29**	May 2022	Yes	BioNTech	4×, 6 wks	F	85
**30**	May 2022	Yes	Moderna	3×, 21 wks	F	59
**31**	May 2022	Yes	BioNTech	3×, 24 wks	F	81

Information about sample collection date, relative to COVID-19 vaccination campaign, injected vaccine, vaccination status, sex, and age of study subjects. ─, not applicable. unk, unknown.

To identify SARS-CoV-2-reactive memory CD4^+^ T cells (31), we determined the frequencies of memory CD4^+^ T cells reacting to a mixture of peptides of the S, M, and N proteins of SARS-CoV-2 in both bone marrow and blood of each individuals (**Figure 1A**). For this, bone marrow and blood mononuclear cells were stimulated with the S/M/N peptides and anti-CD28 for 12 h, and reactive CD4^+^ T cells were identified according to the expression of the activation-induced marker CD154 (32, 33), and one or more of the cytokines IFN-γ, IL-2, and TNF-α, as assessed by intracellular immunofluorescence (**Figure 1D**). Frequencies of cells reacting to stimulation with “anti-CD28 alone”, i.e. the background control, were subtracted from the frequencies of cells reacting to “anti-CD28 plus antigen”. The superantigen SEB, and alternatively CMV-pp65 or tetanus toxoid (TT) were included as controls for stimulation (**Supplemental Figures 1A, B** and data not shown).

CD154^+^cytokine^+^ cells reacting to SARS-CoV-2 were detectable in the blood of 15 donors and in bone marrow of all 17 immunologically naïve donors, at median frequencies significantly higher in bone marrow than in blood, 0.256% (IQR, 0.055%-0.516%) *vs* 0.071% (IQR, 0.018%-0-091%) (*p*<0.01) (**Supplemental Figure 1B**). Interestingly, in two of the unexposed donors, SARS-CoV-2-reactive memory CD4^+^ T cells were only detectable in bone marrow but not in blood (**Supplemental Figure 1B**; highlighted), at the detection limit of 10^-4^ among memory CD4^+^ T cells. In vaccinated donors, frequencies of SARS-CoV-2 reactive CD4^+^CD154^+^cytokine^+^ T cells were higher in bone marrow than in blood in 5 of 14 donors (**Supplemental Figure 1B**).

Based on the frequencies of gated populations and the reasonable estimate that the number of T cells in the bone marrow is 25 × 10^9^, compared to 7 × 10^9^ in the blood (29, 30), human bone marrow of unexposed individuals thus hosts in average (median) sixteen (IQR, 9-13) times more SARS-CoV-2-reactive memory CD4^+^ T cells than blood (*p*<0.0001) (**Figure 1E**), roughly 1.4 × 10^7^ cells. Compared to this, only about three times more SARS-CoV-2-reactive memory CD4^+^ T cells were found in bone marrow than in blood in 7 of 14 vaccinated donors analyzed (**Figure 1E**). Three vaccinated donors did not show a dominant SARS-CoV-2-reactive memory CD4^+^ T cell pool of the bone marrow (**Figure 1E**).

### Bone marrow SARS-CoV-2 reactive memory CD4^+^ T cells are polyfunctional

We next compared the functional imprinting of SARS-CoV-2-reactive memory CD4^+^ T cells from bone marrow and blood. Both frequencies (**Supplemental Figure 1C**) and absolute numbers (**Figure 1F**) of SARS-CoV-2-reactive memory CD4^+^ T cells expressing IFN-γ, TNF-α or IL-2 were significantly higher for bone marrow as compared to blood of unexposed donors. For example, IL-2-producers were detected at median frequencies of 0.207% (IQR, 0.025%-0.276%) for bone marrow versus 0.062% (IQR, 0.004%-0.086%) for blood (**Supplemental Figure 1C**). Median absolute cell numbers were 1.1 ×10^7^ for bone marrow versus 8.2 × 10^5^ for blood (**Figure 1F**). In the 14 vaccinated donors analyzed, absolute numbers (**Figure 1F**) of SARS-CoV-2-reactive memory T cells expressing each of these three cytokines from bone marrow were significantly higher in the bone marrow than in blood, although not in terms of frequencies in most donors (**Supplemental Figure 1C**).

When comparing cells expressing only one of the cytokines analyzed, or two or even all three, all were significantly more abundant among bone marrow cells than those of blood from unexposed donors, except for T cells producing only TNF-α (**Figure 1G**). In terms of frequencies, the cells coexpressing two or three of the cytokines were significantly higher among bone marrow cells than those of blood (**Supplemental Figure 1D**). For vaccinated donors, IFN-γ^+^ single-, IFN-γ^+^TNF-α^+^ double-, IL-2^+^TNF-α^+^ double-, and triple-producers were significantly more abundant among bone marrow memory T cells than among memory T cells of the blood (**Figure 1G**). In terms of frequencies, only IFN-γ^+^ single-producers were significant more frequent among bone marrow cells than those of blood (**Supplemental Figure 1D**).

With considerable individual variation (**Figure 1H**), polyfunctional SARS-CoV-2 reactive memory T cells, i.e. those imprinted to reexpress two or three of the cytokines IL-2, IFN-γ, and TNF-α, were significantly more abundant among bone marrow cells than among blood cells, both in naïve (2.8 × 10^6^ [IQR, 0.6-4.2 × 10^6^] *vs* 1.1 × 10^5^ [IQR, 0.4-3.1 × 10^5^]) and vaccinated donors (4.9 × 10^6^ [IQR, 2.2-21 × 10^6^] *vs* 1.9 × 10^6^ [IQR, 1.0-5.5 × 10^6^]) (**Figure 1I**). The demonstration of those cells even in donors 4 and 7, who did not have detectable numbers of such cells circulating in the blood, implies that those cells are CD4^+^ Trm of bone marrow.

Taken together, our results demonstrate a significant population of polyfunctional, SARS-CoV-2-reactive memory CD4^+^ Trm in the bone marrow in all 17 naïve and 14 vaccinated individuals analyzed.

### Composition of bone marrow cytokine-expressing SARS-CoV-2-reactive memory CD4^+^ T cells in vaccinated donors

Among our vaccinees, SARS-CoV-2-reactive memory T cell responses did not shown patterns related to number of vaccinations received (data now shown). In addition, between our two subcohorts, the age and sex of naïve and vaccinated donors were comparable (**Table 1**). We thus could fairly compare the composition of SARS-CoV-2-reactive memory CD4^+^ T cells from blood and bone marrow with respect to their cytokine expression upon restimulation, i.e. their functional potential. For blood, both frequencies (**Supplemental Figure 1E**) and absolute cell numbers (**Figure 1J**) of the sum of double producers and triple producers, expressing TNF-α (**Supplemental Figure 1F**), were significantly increased in vaccinated donors, in line with the significant increase in total numbers of SARS-CoV-2-reactive cells (**Supplemental Figure 1E** and **Figure 1J**). For bone marrow, a significant decrease in single IL-2-producers was observed, in terms of both frequencies (**Supplemental Figure 1E**) and cell numbers (**Figure 1J**). Numbers of triple producers were significantly increased in bone marrow of vaccinated donors (**Figure 1J**), although they were not in terms of frequencies (**Supplemental Figure 1E**). Overall, we detected about equal absolute numbers and frequencies of bone marrow SARS-CoV-2-reactive cells in naïve and vaccinated donors, with the exception of cells expressing only IL-2 (**Figure 1J** and **Supplemental Figures 1E, F**). Taken together, these results demonstrate an effect of COVID-19 vaccines on reshaping the composition of bone marrow SARS-CoV-2-reactive memory CD4^+^ T cells, consistent with mobilization and participation of CD4^+^ Trm of bone marrow, especially those expressing only IL-2 upon restimulation, in the vaccine-induced immune reaction.

### SARS-CoV-2-reactive central memory CD4^+^ T cells of bone marrow

**Figure 2:**
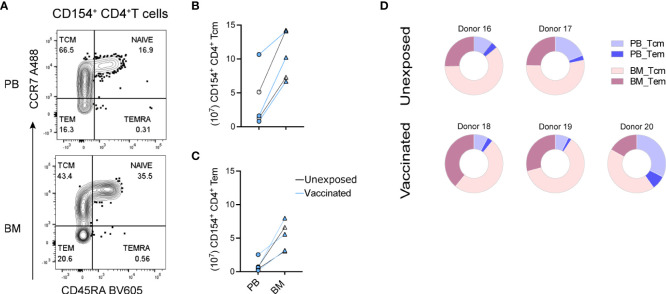
Central memory SARS-CoV-2-reactive memory CD4^+^ T cells of bone marrow. Paired PBMCs and BMMCs from two naïve and three vaccinated donors stimulated with S/M/N of SARS-CoV-2 and analyzed for SARS-CoV-2-reactive CD154^+^ CD4^+^ T cells. **(A)** Representative flow cytometric plots showing their counter expressions for CD45RA and CCR7 on CD154^+^ CD4^+^ T cells (**Supplemental Figure 2A**). (**B + C**) Estimated absolute numbers of CD45RA^-^CCR7^+^ Tcm **(B)** and CD45RA^-^CCR7^-^ Tem **(C)** types of SARS-CoV-2-reactive CD154^+^ memory CD4^+^ T cells of PB and BM, calculated using the frequency (**Supplemental Figures 2B, C**) of these cells and the estimated numbers of T cells within BM and PB as described in **Figure 1**. Blue symbols represent data obtained from vaccinated donors. **(D)** Pie charts showing the proportion of SARS-CoV-2-reactive CD154^+^ memory CD4^+^ T cells in the sum of PB and BM as well as their distribution to Tcm and Tem compartments.

We next determined whether the preexisting SARS-CoV-2-reactive CD154^+^ memory CD4^+^ T cells would resemble central memory T (Tcm) cells, expressing C-C chemokine receptor 7 (CCR7), with the potential to participate in secondary immune reactions in germinal centers, as opposed to CCR7^-^ effector memory T (Tem) cells. SARS-CoV-2-reactive CD154^+^ CD4^+^ T cells (**Supplemental Figure 2A**) were stained for their expression of CCR7 and CD45RA (**Figure 2A**). In the two naïve and three vaccinated donors analyzed, SARS-CoV-2-reactive CCR7^+^CD45RA^-^ Tcm cells (**Supplemental Figure 2B**) were more frequent than CCR7^-^CD45RA^-^ Tem cells in both bone marrow and blood, ranging from 50-70% (**Supplemental Figure 2C**). In absolute numbers, bone marrow contained up to 1.5 × 10^8^ SARS-CoV-2-reactive CD154^+^ Tcm cells (**Figure 2B**) and up to about 0.8 × 10^8^ Tem cells (**Figure 2C**). Thus, bone marrow of naïve and vaccinated donors contained a prominent population of SARS-CoV-2-reactive Tcm cells (**Figure 2D**).

### SARS-CoV-2 reactive CD69^+^ memory CD4^+^ Trm of bone marrow

**Figure 3:**
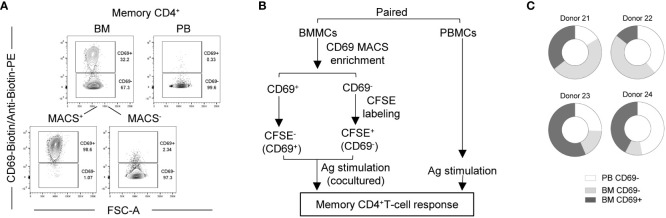
Bone marrow contains CD69^+^ and CD69^-^ SARS-CoV-2 reactive memory CD4^+^ T cells. Bone marrow CD69^+^ and CD69^-^ cells from four vaccinated donors were analyzed for their memory CD4^+^ T cell response to SARS-CoV-2 in comparison with that of paired blood. **(A)** Representative plots showing CD69 expression by memory CD4^+^ T cells from a paired blood and bone marrow samples pre- and/or post-MACS. **(B)** Experimental scheme. **(C)** Estimated absolute numbers of SARS-CoV-2-reactive CD154^+^cytokine^+^ memory CD4^+^ T cells using the frequency (**Supplemental Figure 3C**) of these cells and the estimated numbers of T cells within BM and PB of healthy young adults as described in **Figure 1E**.

CD69 is discussed as a key marker of Trm (34–37), although also CD69^-^ cells can be tissue-resident (22). We thus analyzed the CD69 phenotype of bone marrow SARS-CoV-2-reactive memory CD4^+^ T cells. Because CD69 is upregulated upon activation, we thus isolated CD69^+^ and CD69^-^ cells ex vivo from bone marrow (**Figure 3A**) of 4 vaccinated donors by magnetic cell sorting, labelled the CD69^-^ cells with carboxyfluorescein succinimidyl ester (CFSE) and mixed them with the unlabeled CD69^+^ cells of the same bone marrow (**Supplemental Figure 3A**), and stimulated them with the S/M/N peptides. The reaction of CD69^+^ and CD69^-^ from bone marrow was compared to that of (CD69^-^) memory T cells from their paired blood sample (**Figure 3B**). Both, CD69^+^ and CD69^-^ SARS-CoV-2-reactive CD154^+^cytokine^+^ memory CD4^+^ T cells were readily detectable in the bone marrow (**Supplemental Figure 3B**), and frequencies of either compartment were even higher than that of blood cells in 2 out of the 4 analyzed donors (**Supplemental Figure 3C**). Different to their blood counterparts (>98% being CD69^-^), 30% to 50% of bone marrow memory CD4^+^ T cells of these four vaccinated donors expressed CD69, and thus qualify as bona fide Trm (**Supplemental Figure 3A**). In terms of absolute numbers, these Trm represent 15% to 56% of the sum of blood and bone marrow SARS-CoV-2-reactive memory CD4^+^ T cells (**Figure 3C**). This shows that after vaccination, a substantial number of Trm are maintained in the bone marrow. Whether and if so which CD69^-^ cells are also tissue-resident, remains to be shown. For the preexisting SARS-CoV-2-reactive memory CD4^+^ T cells of naïve individuals described above, the absence of such cells in the circulation of donors 4 and 7 (**Figure 1H**) would argue *per se*, that all their preexisting bone marrow memory T cells are tissue resident, be they CD69^+^ or CD69^-^.

## Discussion

Memory T cells cross-reactive to SARS-CoV-2 S, M, N, and/or envelope peptides have been identified in the peripheral blood of unexposed individuals (1–8,) in 20%-100% of the adult population, depending on the method used to identify them. By their numbers, adapted functional imprinting and fast reaction, these cells have the potential to shape immune reactions to SARS-CoV-2 upon infection of naïve individuals, for the good or for the worse (2–4, 6, 12, 14, 15). Whether and if so which ones could possibly serve as a correlate of protection, is less clear (5). Since the original observation that secondary immune reactions are dependent on tissue-resident, but not on circulating lymphocytes (38), it has become increasingly clear that prominent populations of memory T cells are residing in epithelial tissues, including the lung (39, 40), and also in the bone marrow (17, 41). We have shown that in bone marrow prominent populations of CD4^+^ and CD8^+^ memory T lymphocytes are maintained as Trm, resting individually in niches provided by mesenchymal stromal cells (34, 42). In particular, such memory T cells maintain memory for viral pathogens infecting *via* the airways, but triggering systemic immune reactions, like measles, mumps and rubella (19). We have recently also shown that bone marrow CD4^+^ Trm are mobilized into the blood within a day in secondary immune reactions and contribute significantly to the systemic reaction (27). Here we demonstrate that bone marrow also hosts significant numbers of memory CD4^+^ T cells reactive to SARS-CoV-2, in particular cross-reactive memory T cells preexisting in naïve, SARS-CoV-2 neither infected nor vaccinated individuals.

In all of 17 seronegative, naïve individuals, bone marrow did host SARS-CoV-2 cross-reactive memory CD4^+^ T cells, at absolute numbers of 10^6^ to more than 10^7^, numbers about the same as those of measles-, rubella-, or mumps-specific memory CD4^+^ T cells (19). In 15 out of those 17 naïve individuals, sixteen times more SARS-CoV-2 cross-reactive memory CD4^+^ T cells were present in bone marrow than in blood. In the remaining two naïve individuals, such cells were present only in the bone marrow but not in blood, at a limit of detection of 10^-4^. The presence in bone marrow of all naïve individuals analyzed, of a population of memory CD4^+^ T cells, equal in size to other memories providing long-term immunity to airborne pathogens triggering systemic immune reactions, recapitulates our previous observation for memory T cells reactive to other airborne viruses (19). SARS-CoV-2 obviously is in the same category, and, is mimicked by other antigens, presumably HCoVs, establishing a prominent preexisting population of memory CD4^+^ T cells recognizing SARS-CoV-2 peptides in the bone marrow of naïve individuals. The elevated levels of blood SARS-CoV-2-reactive memory CD4^+^ T cells detected in vaccinated donors are in line with studies showing that higher frequencies of SARS-CoV-2-reactive CD4^+^ T cells could be induced by infection (14) or vaccination (12) from previously unexposed donors, which thereafter show cross-reactive CD4^+^ T cells in the peripheral blood. However, these enhanced numbers of circulating memory T cells upon vaccination are not at the expense of bone marrow memory. Numbers and composition of bone marrow memory CD4^+^ T cells in vaccinated individuals is similar to that of naïve individuals, with the exception of a drop in IL-2 single producers.

Which antigens did prime the SARS-CoV-2-reactive memory T cells in the naïve individuals analyzed? SARS-CoV-2 related viruses, such as endemic HCoVs or SARS have been discussed (5, 7, 9, 10, 31, 43), as well as unrelated viruses, such as CMV, influenza virus, EBV, and adenoviruses. Moreover, even upon measles-mumps-rubella (MMR), or Tetanus-Diphtheria-Pertussis (Tdap) vaccination, generation of cross-reactive T cells that mitigate COVID-19 has been described (44). The present analysis does not provide clues to the original antigenic provocations establishing the SARS-CoV-2 reactive circulating and bone marrow Trm populations, except that it is clear that this cross-reactive memory is ubiquitous, polyfunctional and contains a considerable population of central memory CD4^+^ T cells.

The SARS-CoV-2 cross-reactive memory CD4^+^ T cells in the bone marrow of adult humans are functionally superior to their circulating counterparts, in terms of imprinting for expression of cytokines upon restimulation. Many of them then display an IFN-γ- and IL-2-imprinted, “polyfunctional” Th1 profile. This suggests that bone marrow resident, cross-reactive memory CD4^+^ T cells would provide superior protection, when compared to their circulating counterparts (5). It has been demonstrated that the initial frequency of IL-2 secreting cross-reactive memory T cells is associated with protection of naïve individuals from infection in COVID-19 contacts (4), and SARS-CoV-2-reactive IFN-γ-secreting T cells have been associated with protection from reinfection (45). Notably, polyfunctionality of SARS-CoV-2 spike-reactive CD4^+^ T cells correlates with low severity of COVID-19 (13). In addition to their polyfunctionality, many bone marrow resident SARS-CoV-2-reactive memory CD4^+^ T cells are of the CCR7^+^ Tcm type, indicating the potential of self-renewal, progression into effector cell differentiation, and also qualifying them as helper cells for follicular immune reactions (46, 47). As polyfunctional Tcm cells they resemble SARS-CoV-2 CD4^+^ T cells 12 months post-infection (48). Together with the plethora of data reporting the SARS-CoV-2-reactive, circulating memory CD4^+^ T cells, the present data demonstrate that there is also a preexisting potent population of bone marrow-resident memory CD4^+^ t cells, comparable to those reactive to measles, mumps, or rubella. It becomes evident that SARS-CoV-2 did meet a human population with most adult individuals having a prominent, persistent, polyfunctional cross-reactive immunological memory T-cell population. Thus, vaccination and infection did meet “prepared” immune systems with preexisting systemic CD4^+^ T-cell immunity. Of note, however, the ubiquitous preexisting CD4^+^ T cell memory is not matched with an equally ubiquitous B cell memory (49). Nevertheless, an efficient germinal center reaction is established in this dialog between preexisting memory T cells and newly recruited naïve B cells (50). Since the establishment of immunological memory is considered to depend on signal from CD4^+^ memory T cells, this might have contributed to the impressive establishment of memory plasma cells in the bone marrow of most convalescent or vaccinated individuals, after only one infection or two vaccine encounters 3 to 4 weeks apart (51). It is tempting to speculate that the preexisting T cell memory may also have contributed to the rapid, prominent and obviously protective immune responses to SARS-CoCV-2 in most infected individuals (2–4, 6, 12, 14, 15). Conversely, the few severely affected individuals may have had a no or a malfunctional preexisting CD4^+^ T cell memory (5) (31, 52, 53).

Since access to human bone marrow is limited, the present study is limited in this aspect as well, to a small cohort of 17 naïve and 14 vaccinated individuals. Further analyses of the role of Trm in infection and vaccine responses would benefit from a non-invasive access to these Trm. We have recently shown that bone marrow Trm specific for the MMR vaccine are mobilized within a day into the blood following a secondary MMR vaccination, and contribute essentially to the secondary immune reaction (27). Notably, also cross-reactive Trm, e.g. such recognizing both TT and measles, were mobilized by MMR revaccination (27). Thus, preexisting Trm when reactivated and mobilized into the blood on their way to the secondary systemic immune reaction, can be assessed in the blood on day 1 after challenge, as compared to the circulating cells that are accessible already on day 0.

Our cohort of 17 naïve probands was too small to compare young and aged individuals, with 11 individuals analyzed aged 60 or above, to answer the question whether those bone marrow resident cross-reactive memory T cells have a different phenotype or repertoire or are missing at all in some of them, explaining the retarded reaction to vaccination in some elderly (54) and the enhanced, but still low incidence of severe COVID-19 in the elderly (5). In view of the ubiquitous presence of these cells in all our probands, the existence of experienced single and/or polyfunctional memory T cells cross-reactive and quickly reacting to SARS-CoV-2 might be a decisive advantage in successful immune reactions and moderate to mild COVID-19, also in the elderly, considering the immediate attempt of the virus to evade our immune response, by inducing expression of the immunosuppressive cytokine TGF-β (1, 55, 56).

## Data availability statement

The original contributions presented in the study are included in the article/**Supplementary Material**. Further inquiries can be directed to the corresponding author.

## Ethics statement

This study was reviewed and approved by Ethikkommission der Charité-Univerisitätsmedizin Berlin; EA1/005/21. The patients/participants provided their written informed consent to participate in this study. Written informed consent was obtained from the individual(s) for the publication of any potentially identifiable images or data included in this article.

## Author Contributions

Conceptualization: JD. Methodology: JL, YS, LS, SF, JD. Investigation: JL, YS, LS, ZW. Formal Analysis: JL, YS, LS, YCL, JD. Resources: SR, SH, CH, UH, HDC, TA, CP, HR, ZQ. Interpretation: JL, YS, LS, AR, JD. Visualization: JL, JD. Funding Acquisition: JD, AR. Project Administration: JD. Supervision, JD. Writing – Original Draft, JD. Writing – Review and Editing, AR, JD. All authors contributed to the article and approved the submitted version.

## Funding

Chinesisch-Deutsches Zentrum für Wissenschaftsförderung (CDZ; DFG im Ausland) grant C-0072 (JD). Deutsche Forschungsgemeinschft (DFG) Project 389687267 (JD and AR). Leibniz science Campus Chronic Inflammation (www.chronische-entzuendung.org). HDC was supported by Dr. Rolf M. Schwiete Foundation.

## Acknowledgments

We are thankful to Antje Blankenstein and Johanna Penzlin for organizing patient materials, to Dr. Weijie Du for technical support, to Dr. Liqun He for his comments on data presentation, and to Drs. Alex Schefold, Petra Bacher and Chiara Romagnani for their critical comments on the manuscript/findings. We also want to thank Hilmar Frank for his expert IT solutions to figure preparation.

## Conflict of interest

The authors declare that the research was conducted in the absence of any commercial or financial relationships that could be construed as a potential conflict of interest.

## Publisher’s note

All claims expressed in this article are solely those of the authors and do not necessarily represent those of their affiliated organizations, or those of the publisher, the editors and the reviewers. Any product that may be evaluated in this article, or claim that may be made by its manufacturer, is not guaranteed or endorsed by the publisher.
