# Supplementary material for: A ubiquitous bone marrow reservoir of preexisting SARS-CoV-2-reactive memory CD4+ T lymphocytes in unexposed individuals
Source: Front Immunol. 2022 Oct 4;13:1004656. doi: 10.3389/fimmu.2022.1004656 (PMC9576920; doi:10.3389/fimmu.2022.1004656)
Supplement: Supplementary file 1 [file DataSheet_1.pdf]

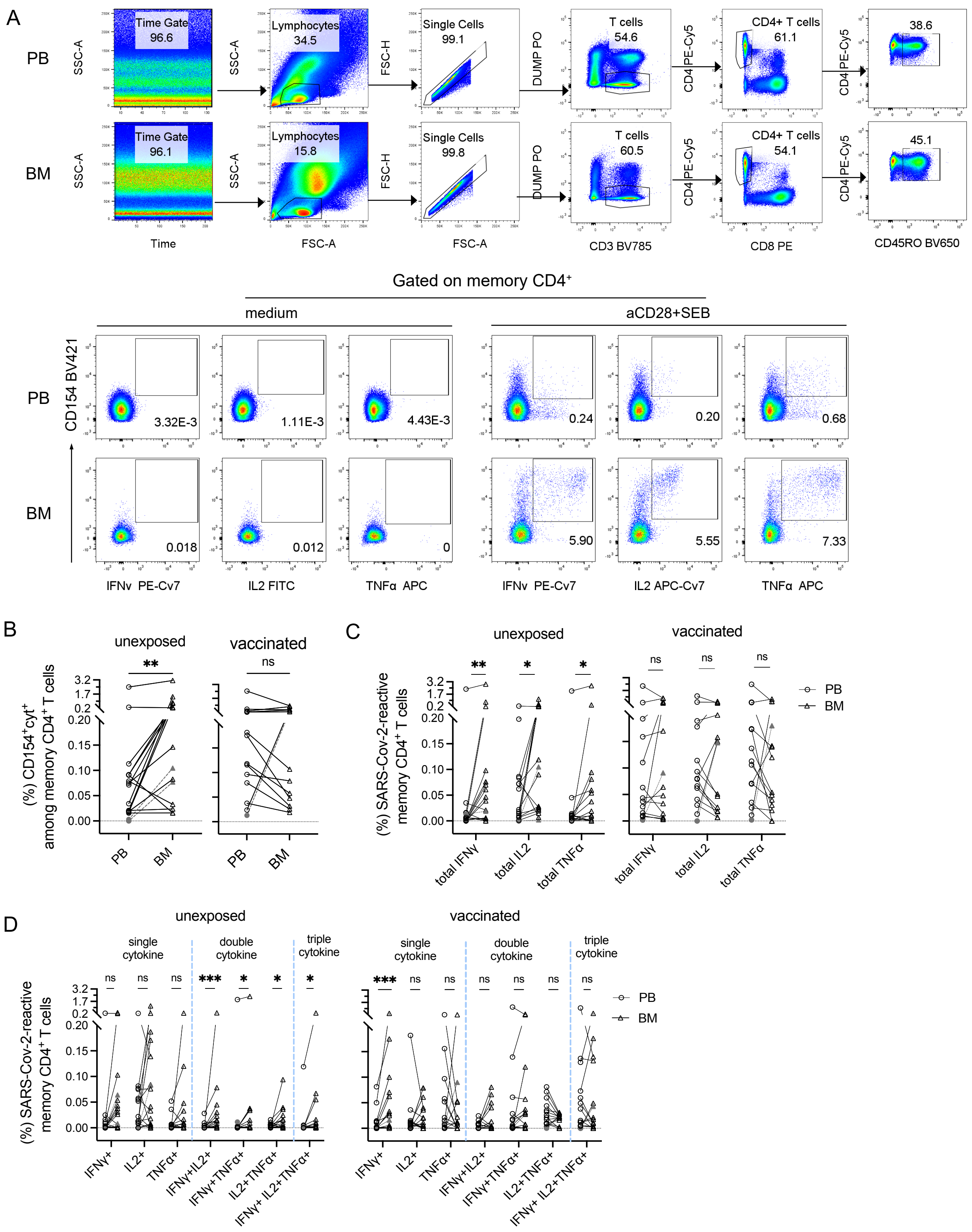

A
PB
BM
Time Gate 96.6
Time Gate 96.1
Lymphocytes 34.5
Lymphocytes 15.8
Single Cells 99.1
Single Cells 99.8
T cells 54.6
T cells 60.5
CD4+ T cells 61.1
CD4+ T cells 54.1
38.6
45.1
SSC-A
FSC-H
DUMP PO
CD4 PE-Cy5
Time
FSC-A
CD3 BV785
CD8 PE
CD45RO BV650
Gated on memory CD4+
medium
aCD28+SEB
CD154 BV421
3.32E-3
1.11E-3
4.43E-3
0.24
0.20
0.68
0.018
0.012
0
5.90
5.55
7.33
IFNγ PE-Cy7
IL2 FITC
TNFα APC
IL2 APC-Cy7
B
unexposed
vaccinated
**
ns
(%) CD154+cyt+ among memory CD4+ T cells
PB
BM
C
*
(%) SARS-Cov-2-reactive memory CD4+ T cells
total IFNγ
total IL2
total TNFα
D
single cytokine
double cytokine
triple cytokine
***
IFNγ+
IL2+
TNFα+
IFNγ+IL2+
IFNγ+TNFα+
IL2+TNFα+
IFNγ+ IL2+TNFα+

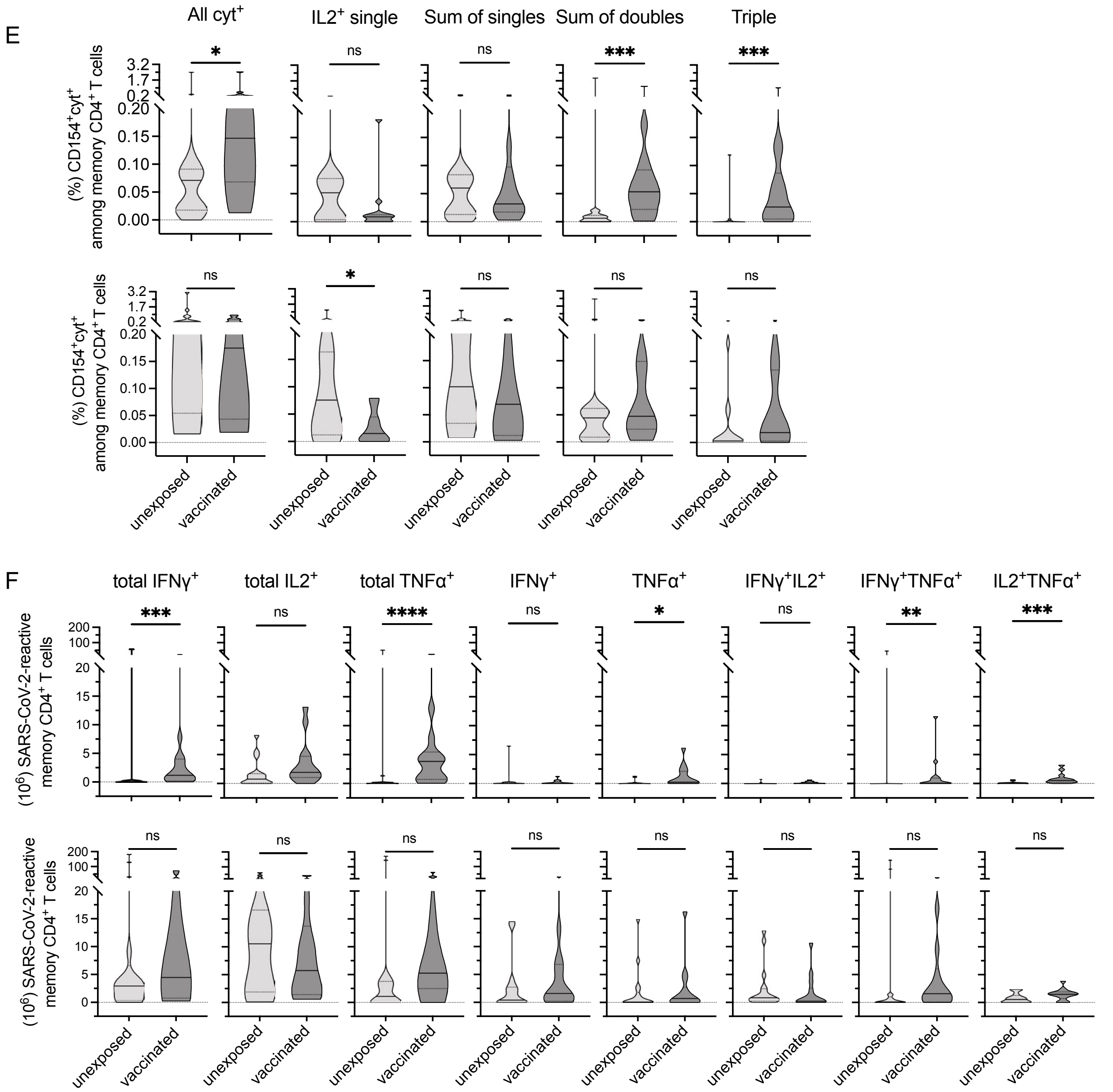

Supplemental Figure 1. Analysis of SARS-CoV-2-reactive memory CD4+ T cells from paired blood and bone marrow samples, Related to Figure 1. Paired PBMCs and BMMCs from unexposed (n = 17) and COVID-19 vaccinated (n = 14) donors were stimulated with antigen, and the induced cytokine production (IL-2, TNF-α, and/or IFN-γ) in live memory CD4+ T cells was analyzed according to CD154 expression by intracellular cytokine staining. (A) Gating strategy. DUMP channel included fluorophore staining for dead cells as well as CD19+ and CD14+ cells non CD3+ T cells. Cytokines from gated BM and PB memory CD4+ T cells cultured in medium alone and induced by the high control SEB stimulation are shown. (B) Frequencies of CD154+cytokine+ memory CD4+ T cells from PB and BM. Boolean gating approach was applied to generate results for all possible cytokine combinations. For each subpopulation, the background (as detected in the anti-CD28 stimulated control samples) was subtracted. Symbols in grey and dashed lines indicate frequencies under reliable detection limit (10^{-4} of memory CD4+ T cells). (C) Frequencies of total individual cytokine producing cells. (D) Frequencies of single, double and triple cytokine producers. (E, F) Comparison of frequencies (E) and estimated absolute cell numbers (F) of indicated populations in between unexposed and vaccinated donors. Values are presented as median (thick line) with 25th- and 75th-percentile. Statistical significance between paired samples and unpaired samples were calculated based on Wilcoxon matched-pairs signed ranks test and Mann-Whitney U test, respectively. *$p<0.05$, **$p<0.01$, ***$p<0.001$, ****$p<0.0001$; ns, not significant.

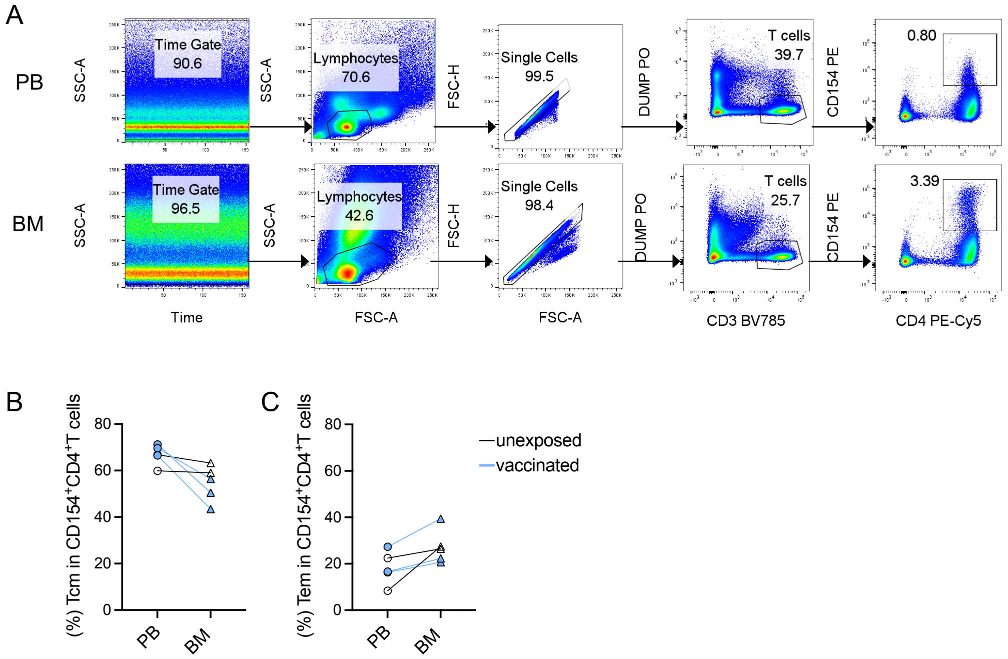

Supplemental Figure 2. Tcm and Tem composition of SARS-CoV-2-reactive memory CD4^{+} T cells from paired blood and bone marrow samples, Related to Figure 2. Paired PBMCs and BMMCs from two unexposed and three COVID-19 vaccinated individuals were analyzed for their induced SARS-CoV-2-reactive CD154^{+} CD4^{+} T cells (A) for analyzing their expression of CD45RA and CCR7. (B, C) Frequencies of Tcm (CD45RA^{-}CCR7^{+}; B) and Tem (CD45RA^{-}CCR7^{-}; C) subsets among the SARS-CoV-2-reactive CD154^{+} memory CD4^{+} T cells. Tcm, central memory T cells; Tem, effector memory T cells.

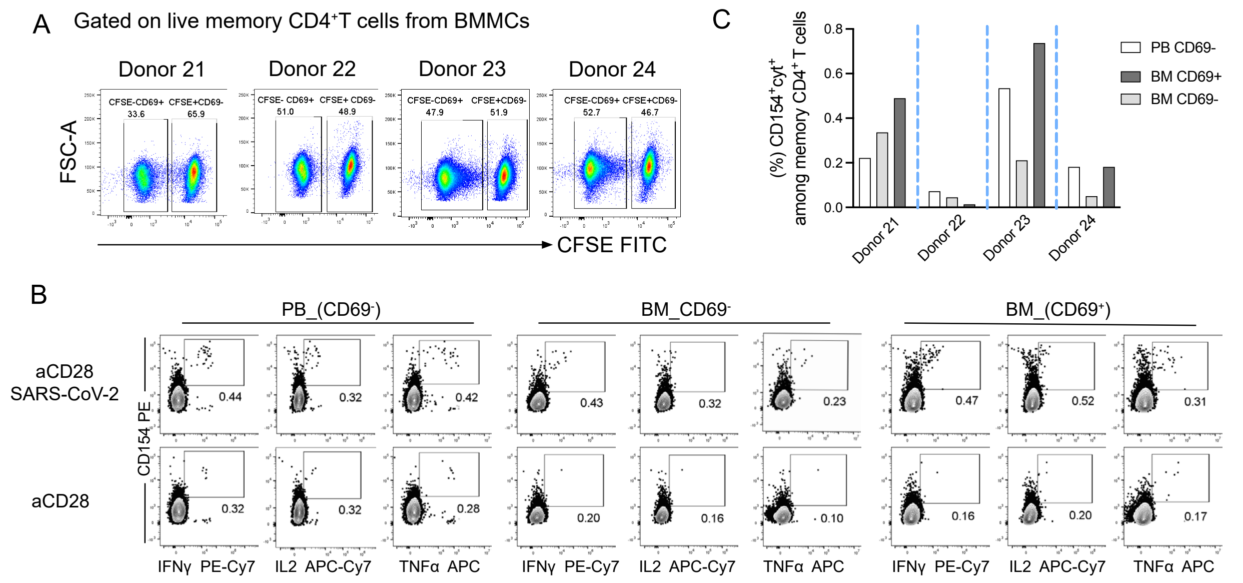

Supplemental Figure 3. Compartmentalization of bone marrow SARS-CoV-2-reactive CD4$^+$ memory T cells, Related to Figure 3. Bone marrow CD69$^+$ and CD69$^-$ cells from four vaccinated donors were analyzed for their memory CD4$^+$ T cell response to SARS-CoV-2 in comparison with that of paired blood. CD69$^+$ cells from BMMCs were magnetically enriched and the resulting CD69$^-$ fraction was labeled with CFSE and then both fractions were co-cultured for stimulation. (A) Flow plots showing the separation of bone marrow memory CD69$^+$ cells from CD69$^-$ cells. (B) Representative flow plots showing "anti-CD28" and "anti-CD28 plus SARS-CoV-2 S/M/N antigenmix" induced cytokine productions IL-2, TNF-α, and IFN-γ according to CD154 expression by intracellular cytokine staining. (C) Frequencies of the induced cytokine production (IL-2, TNF-α, and/or IFN-γ) in SARS-CoV-2-reactive CD154$^+$cytokine$^+$memory CD4$^+$ T cells among bone marrow CD69$^+$ and CD69$^-$ compartments and in direct relation to those detected in their paired blood. (D) Estimated absolute cell numbers of SARS-CoV-2-reactive CD154$^+$ memory CD4$^+$ T cells in paired blood and bone marrow including their proportions in CD69$^+$ and CD69$^-$ subsets are shown.
