# Supplementary material for: A ubiquitous bone marrow reservoir of preexisting SARS-CoV-2-reactive memory CD4+ T lymphocytes in unexposed individuals
Source: Front Immunol. 2022 Oct 4;13:1004656. doi: 10.3389/fimmu.2022.1004656 (PMC9576920; doi:10.3389/fimmu.2022.1004656)
Supplement: Supplementary file 2 [file Table_1.docx]

**Supplemental Table. Key resources table**

| **Reagent or Resource** | **Source** | **Identifier** |
| --- | --- | --- |
| **Antibodies (concentration)** | | |
| anti-CD3 BV785 (1:100) | Biolegend | Cat# 317330;  RRID: AB_2563507 |
| anti-CD4 PE-Cy5 (1:200) | Biolegend | Cat# 300510;  RRID: AB_314078 |
| anti-CD8 PE (1:100) | In house | Clone GN11/B4D7 |
| anti-CD14 PO (1:100) | In house | Clone TM1 |
| anti-CD19 BV510 (1:100) | Biolegend | Cat# 302242;  RRID: AB_ 2561668 |
| anti-CD45RA BV605 (1:100) | Biolegend | Cat# 304134;  RRID: AB_2563814 |
| anti-CD45RO BV650 (1:100) | Biolegend | Cat# 304232;  RRID: AB_2563462 |
| anti-CCR7 A488 (1:100) | Biolegend | Cat# 353206;  RRID: AB_10916389 |
| anti-CD154 BV421 (1:100) | Biolegend | Cat# 310824;  RRID: AB_2562721 |
| anti-CD154 PE (1:100) | Miltenyi Biotec | Cat# 130-113-613 |
| anti-TNFα APC (1:100) | BD Pharminogen | Cat# 551384 |
| anti-IFNγ PE-Cy7 (1:200) | Biolegend | Cat# 502528;  RRID: AB_2123323 |
| anti-IL-2 FITC (1:100) | Biolegend | Cat# 500304; RRID: AB_315091 |
| anti-IL-2 APC-Cy7 (1:100) | Biolegend | Cat# 500342;  RRID: AB_2562855 |

| **Chemicals, peptides, proteins, and others** | | |
| --- | --- | --- |
| Ficoll-Paque Plus | Cytiva | Cat# 17-1440-03 |
| Buffer EL | Qiagen | Cat# 79217 |
| Formaldehyde solution | Carl Roth | Cat# 4979.3 |
| BD FACS Permeabilizing solution 2 | BD Biosciences | Cat# 347692 |
| Brefeldin A | Biolegend | Cat# 420601 |
| CD28 pure - functional grade | Miltenyi Biotec | Cat# 130-093-375 |
| FcR blocking reagent | Miltenyi Biotec | Cat# 130-059-901 |
| CMV-pp65 | Miltenyi Biotec | Cat# 130-091-824 |
| Tetanus Toxoid | NIBSC | Cat# 02/232 |
| Staphylococcus enterotoxin B | Sigma-Aldrich | Cat# S4881-1MG |
| PepTivator^®^ SARS-CoV-2 Prot_M | Miltenyi Biotec | Cat# 130-126-702 |
| PepTivator^®^ SARS-CoV-2 Prot_N | Miltenyi Biotec | Cat# 130-126-698 |
| PepTivator^®^ SARS-CoV-2 Prot_S | Miltenyi Biotec | Cat# 130-126-700 |
| Human male Ab serum | Sigma-Aldrich | Cat# H4522-100ML |
| CellTrace™ CFSE Cell Proliferation Kit (final conc. 2 nM) | ThermoFischer | Cat# C34554 |
| Zombie Aqua™ Fixable Viability Kit (Live/Dead; Pacific Orange, 1:400) | Biolegend | Cat# 423101 |
| Penicillin-Streptomycin-Glutamine (100X) | ThermoFischer | Cat# 10378016 |
| RPMI Medium 1640 - GlutaMax | ThermoFischer | Cat# 21875-091 |
| LS magnetic separation columns | Miltenyi Biotec | Cat# 130-042-401 |
| MS magnetic separation columns | Miltenyi Biotec | Cat# 130-042-201 |
| Pre-separation filters, 30 μm | Miltenyi Biotec | Cat# 130-041-407 |
| LEGEND MAX^TM^ ELISA kit SARS-CoV-2 Nucleocapsid Human IgG | Biolegend | Cat# 448107 |
| LEGEND MAX^TM^ ELISA kit SARS-CoV-2 Spike S1 Human IgG | Biolegend | Cat# 447807 |
| CD69 MicroBead Kit II | Miltenyi Biotec | Cat# 130-092-355 |
| **Softwares** | | |
| Flowjo LLC v9/10 | BD Biosciences |  |
| GraphPad Prism 9 | GraphPad Software |  |
| EndNote X9 | Endnote^TM^ |  |
